# Clinical Outcomes of Plasma-Assisted Saline Irrigation in Nonsurgical Root Canal Treatment: A Preliminary Retrospective Cohort Study

**DOI:** 10.3390/biomedicines14061389

**Published:** 2026-06-19

**Authors:** Young-Hee Kim, Jeong-Hyo Lyu, Hyun-Sook Chung, Sang-Yoon Park, Sang-Min Yi, Soo-Hwan Byun, Sung-Woon On, Jae-Seo Lee, Dong-Jun Kim, Byoung-Eun Yang

**Affiliations:** 1 Division of Oral and Maxillofacial Radiology, Hallym University Sacred Heart Hospital, Anyang 14066, Republic of Korea; kcallas2@gmail.com; 2 Department of Artificial Intelligence and Robotics in Dentistry, Graduate School of Clinical Dentistry, Hallym University, Chuncheon 24252, Republic of Korea; lyujh0519@naver.com (J.-H.L.); dnendo@gmail.com (H.-S.C.); psypjy0112@naver.com (S.-Y.P.); queen21c@hallym.or.kr (S.-M.Y.); purheit@daum.net (S.-H.B.); drummer0908@hanmail.net (S.-W.O.); 3 Institute of Clinical Dentistry, Hallym University, Chuncheon 24252, Republic of Korea; 4 Dental Artificial Intelligence and Robotics R&D Center, Hallym University Medical Center, Anyang 14066, Republic of Korea; 5 Division of Conservative Dentistry, Hallym University Sacred Heart Hospital, Anyang 14066, Republic of Korea; 6 Department of Oral and Maxillofacial Radiology, School of Dentistry, Chonnam National University, Gwangju 61186, Republic of Korea; jsyi16@jnu.ac.kr; 7 Kim Dong-Jun Dental Clinic, Gwangju 61940, Republic of Korea; conskim94@gmail.com

**Keywords:** underwater discharge plasma, non-thermal plasma, root canal treatment outcomes, apical periodontitis, periapical healing

## Abstract

**Background:** Effective root canal disinfection is essential for successful nonsurgical root canal treatment (RCT). Although sodium hypochlorite (NaOCl) remains the standard irrigant, it carries a risk of chemical tissue injury if extruded beyond the root canal system and may have limited penetration into anatomically complex regions. Underwater discharge plasma (UDP) generates reactive oxygen and nitrogen species (RONS) through high-frequency, high-voltage electrical discharge in aqueous media, and preclinical and in vitro studies have reported broad-spectrum antimicrobial activity. This study evaluated the clinical and radiographic outcomes of nonsurgical RCT performed using physiological saline-based UDP irrigation without NaOCl in a heterogeneous real-world clinical cohort. **Methods:** This single-center retrospective cohort study included 186 teeth from 134 patients treated with the PLAZEN RCT^®^ UDP device and physiological saline irrigation, without NaOCl. The median follow-up period was 16 months. Radiographic outcomes were assessed using the Periapical Index (PAI) system, and treatment success was evaluated according to prespecified Strict and Loose criteria incorporating both radiographic and clinical findings. Stratified analysis was performed according to preoperative PAI score: Group A (PAI 1–2) and Group B (PAI 3–5). UDP-related adverse events, defined as thermal tissue injury caused by discharge heat, were ascertained through retrospective review of clinical records, operative notes, and serial periapical radiographs. **Results:** Among the 186 treated teeth, radiographic outcomes were classified as Healed (85.5%), Healing (3.8%), and Unhealed (10.8%). Overall Strict and Loose success rates were 79.6% and 82.3%, respectively. Initial treatment showed numerically higher success rates than retreatment. In the stratified analysis, Group A showed an 84.1% success rate with 100% tooth survival, whereas Group B demonstrated Strict and Loose success rates of 68.5% and 83.3%, respectively. Exploratory multivariable analysis showed that periodontal pocket depth > 3 mm was the most consistent factor associated with lower odds of treatment success, whereas associations involving canal obliteration and higher preoperative PAI score were less stable across sensitivity analyses and should be interpreted with caution. No UDP-related adverse events were recorded during follow-up. Attrition sensitivity analyses were performed, and the outcome estimates should be interpreted with caution, given the retrospective design and substantial loss to follow-up. **Conclusions:** In this preliminary observational cohort, physiological saline-based UDP irrigation without NaOCl was associated with favorable observed periapical healing outcomes and no recorded UDP-related adverse events over a median follow-up of 16 months. However, loss to follow-up was substantial; when all 116 teeth lost to follow-up were classified as treatment failures, the worst-case Strict success rate decreased to 49.0%. Therefore, these findings should be interpreted as preliminary descriptive evidence of clinical feasibility rather than as evidence of comparative efficacy or definitive clinical safety. Adequately powered randomized controlled trials with concurrent NaOCl control arms and long-term follow-up are warranted to evaluate the comparative effectiveness, safety, and reproducibility of physiological saline-based UDP irrigation protocols.

## 1. Introduction

Despite continued advances in root canal disinfection protocols, persistent apical disease after nonsurgical root canal treatment (RCT) remains a clinically relevant problem [1,2]. This underscores the need for irrigation strategies that combine antimicrobial activity with acceptable tissue compatibility. Sodium hypochlorite (NaOCl), the current standard irrigant, possesses excellent antimicrobial and tissue-dissolving properties; however, inadvertent extrusion beyond the root apex can cause severe soft-tissue injury and other complications [3]. In addition, NaOCl has been recognized as having limitations in eliminating biofilms and organic debris from anatomically complex regions of the root canal system, including lateral canals, isthmuses, and irregular canal extensions [4,5]. A recent umbrella review synthesizing systematic reviews and meta-analyses on irrigation protocols in endodontic therapy identified substantial variability among irrigants and protocols, persistent methodological limitations in the current evidence base, and an absence of standardized evidence-based clinical guidelines for root canal disinfection [6], supporting the need for rigorous clinical investigation of alternative or adjunctive irrigation approaches.

To address these challenges, several irrigation activation technologies have been developed and clinically evaluated. Passive ultrasonic irrigation enhances irrigant penetration through acoustic streaming and cavitation and has been shown to improve root canal disinfection and debris removal compared with conventional syringe irrigation [7]. Sonic activation systems, such as EDDY, employ flexible polymer tips that generate three-dimensional fluid motion, reducing the risk of mechanical damage to the canal wall and offering a complementary approach to conventional ultrasonic methods [8]. Laser-activated irrigation, including photon-induced photoacoustic streaming and Er: YAG laser systems, generates shockwaves and fluid agitation capable of disrupting biofilms in anatomically complex regions; however, concerns about irrigant extrusion beyond the apical foramen and equipment cost have limited its widespread clinical adoption [9]. Multisonic irrigation systems, such as GentleWave, utilize broad-spectrum acoustic energy to achieve full-length canal debridement while preserving tooth structure; however, they are commonly used with NaOCl as the primary irrigant and therefore retain the safety considerations associated with hypochlorite-based chemistry [10]. Despite the advances offered by these activation technologies, many clinically established approaches remain centered on NaOCl-based disinfection. This supports the need to investigate alternative disinfection strategies that may reduce dependence on NaOCl-based chemistry while maintaining clinically acceptable outcomes.

**Figure 1:**
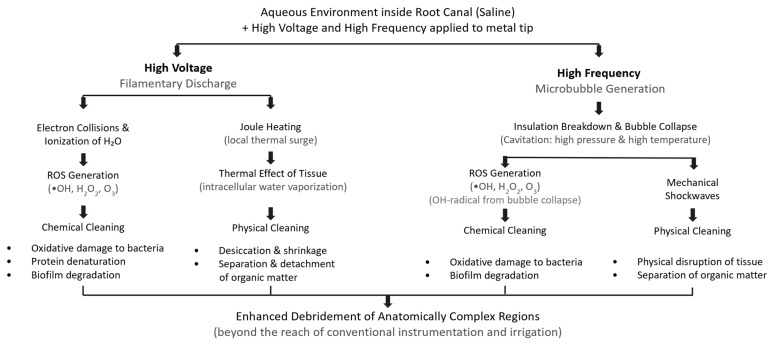
Conceptual schematic of the proposed chemical and physical mechanisms of underwater discharge plasma (UDP) during root canal irrigation. The mechanisms illustrated, including high-voltage discharge in an aqueous environment, microbubble formation and cavitation, generation of reactive oxygen and nitrogen species (RONS) such as hydroxyl radicals (•OH), hydrogen peroxide (H_2_O_2_), and ozone (O_3_), and mechanical fluid agitation or shockwave generation, are extrapolated from prior experimental studies of high-frequency electrical discharge in aqueous media and non-thermal plasma chemistry [11,16,17,18,19,24]. These mechanisms were not directly validated for the PLAZEN RCT^®^ UDP device during endodontic irrigation in the present study. The figure is provided for conceptual illustration only. RONS = reactive oxygen and nitrogen species; UDP = underwater discharge plasma.

Non-thermal plasma (NTP) has been investigated as a potential antimicrobial modality, based on broad-spectrum antimicrobial activity demonstrated in preclinical and in vitro studies, along with reported biocompatibility [11,12]. Underwater discharge plasma (UDP), a subtype of NTP, generates plasma directly within an aqueous medium and has been investigated for its antibiofilm and antimicrobial potential in endodontic disinfection [13,14,15]. In the context of the present study, plasma-assisted saline irrigation refers specifically to physiological saline-based UDP irrigation, in which plasma discharge is activated within a saline-filled root canal without the use of NaOCl. Thus, UDP is used as an activation modality within the irrigating medium rather than as a conventional liquid irrigant. By generating reactive oxygen and nitrogen species (RONS) and associated fluid-dynamic effects, UDP may facilitate antimicrobial effects within anatomically complex regions, such as lateral canals and isthmuses, that are otherwise difficult to access with conventional chemical irrigants [16,17,18] (Figure 1).

The clinical feasibility of UDP was recently reported in a prospective randomized pilot trial by Lyu et al. [19]. However, the limited sample size and short follow-up period of that study (4 months) precluded evaluation of longer-term clinical and radiographic outcomes. Because periapical healing is a dynamic process that may continue for several years [20,21], the European Society of Endodontology guidelines recommend assessing treatment outcomes at least 12 months after obturation [22].

Accordingly, this retrospective cohort study aimed to evaluate the clinical and radiographic outcomes of nonsurgical RCT performed using physiological saline-based UDP irrigation in a heterogeneous real-world clinical cohort. A stratified analysis based on preoperative periapical status was also performed to account for differences in baseline disease severity [23].

## 2. Materials and Methods

### 2.1. Study Design and Ethical Approval

This single-center retrospective cohort study was designed to evaluate the short- to medium-term clinical and radiographic outcomes of nonsurgical root canal treatment (RCT) performed using physiological saline-based underwater discharge plasma (UDP) irrigation. The study was conducted in accordance with the principles of the Declaration of Helsinki and was approved by the Institutional Review Board (IRB) of Hallym University Sacred Heart Hospital (IRB No. 2026-03-032-001). The requirement for individual informed consent was waived because of the retrospective medical record review design. All data were anonymized prior to analysis to protect patient confidentiality.

The study size was determined pragmatically. No a priori sample size or precision calculation was performed, and the analytical cohort comprised all consecutive eligible teeth treated with the PLAZEN RCT^®^ UDP device (Dentory, Seoul, Republic of Korea) during the defined study period from July 2023 to March 2026. As a post hoc assessment of precision, the observed Strict success rate of 79.6% was estimated with a 95% confidence interval of 73.1–85.0%, providing a quantitative indication of estimate precision for the primary descriptive outcome estimate. This study did not include a concurrent NaOCl control group, which substantially limits any interpretation of comparative efficacy, as discussed in Section 4.

The present retrospective cohort study was conducted after a preceding prospective randomized pilot trial of UDP-based irrigation [19]. The two studies involved an overlapping author group: eight of the nine authors of the present study also contributed to the predecessor pilot trial. The same PLAZEN RCT^®^ UDP device, physiological saline-based UDP protocol, and experienced operator were used. However, the present study differs from the predecessor trial in study design, follow-up duration, analyzed cohort, follow-up outcome data, and adjudication of outcomes. The predecessor trial reported short-term clinical feasibility and adverse-event observations with a maximum follow-up of 4 months, whereas the present study evaluated longer-term clinical and radiographic outcomes over 12–30 months. In the present study, radiographic outcome assessment was independently performed by two external examiners who were blinded to clinical information, treatment details, UDP application parameters, and predefined clinical success/failure classifications.

Of the 186 teeth included in the present cohort, 11 originated from patients who had been enrolled in the UDP arm of the predecessor pilot trial [19]. These teeth were included in the present study only because they met the prespecified eligibility criteria for the present retrospective cohort, including a minimum follow-up duration of 12 months. The predecessor trial reported outcomes only up to 4 months; therefore, no follow-up outcomes from that trial were duplicated in the present analysis. Teeth from UDP-treated pilot-trial participants that did not meet the 12-month minimum follow-up criterion were not included in the present cohort.

### 2.2. Eligibility Criteria

Electronic medical records of 341 teeth from 229 patients who underwent nonsurgical RCT using the PLAZEN RCT^®^ UDP device at the Department of Conservative Dentistry, Hallym University Sacred Heart Hospital, between July 2023 and March 2026 were retrospectively reviewed. The tooth was defined as the unit of analysis. The study period commenced in July 2023, coinciding with the first clinical introduction of the PLAZEN RCT^®^ UDP device at our institution. All teeth treated with this device from its introduction through the database lock date of 31 March 2026 were consecutively identified through a systematic search of electronic medical records; no case selection beyond the prespecified eligibility criteria was applied. Of these, 155 teeth were excluded according to the eligibility criteria, yielding a final analytical cohort of 186 teeth from 134 patients.

The inclusion criteria were as follows: (1) nonsurgical RCT performed using the standardized physiological saline-based UDP protocol without NaOCl, derived from that reported by Lyu et al. [19]; (2) availability of preoperative periapical radiographs; and (3) adequate radiographic image quality for Periapical Index (PAI) scoring.

The exclusion criteria were as follows: (1) insufficient pre- or post-treatment radiographic records; (2) follow-up duration of less than 12 months after obturation; (3) use of mixed irrigants, NaOCl, or additional disinfection methods other than the standardized UDP protocol; (4) tooth extraction for reasons unrelated to endodontic failure; (5) death of the patient during the follow-up period before outcome assessment; and (6) root canal treatment performed prior to planned cyst enucleation, because the treatment objective differed from that of conventional nonsurgical endodontic therapy.

Regarding criterion (4), all five teeth extracted during the follow-up period were excluded because the extraction was attributable to vertical root fracture (VRF), as documented in the clinical records. VRF was considered a non-endodontic cause of tooth loss in the present study because it represents a mechanical complication rather than persistent or progressive periapical pathology. No teeth were extracted due to endodontic treatment failure during follow-up.

The 12-month minimum follow-up threshold was applied in accordance with the ESE quality guidelines, which recommend that endodontic treatment outcomes be assessed at least 12 months after obturation [22], as periapical healing is a dynamic process that may take 1 or more years to complete [20]. The 18 teeth excluded due to the follow-up duration of less than 12 months, including cases with 10 or 11 months of follow-up at their last recorded visit, represented loss from observation before the minimum outcome assessment time point: their last recorded clinical contact occurred before 12 months with no subsequent follow-up visit thereafter. These were not cases of active follow-up administratively curtailed by the database lock date of 31 March 2026.

### 2.3. Root Canal Treatment and UDP Application Protocol

All root canal treatment procedures were performed by a single experienced operator using a standardized physiological saline-based UDP protocol derived from the predecessor pilot trial by Lyu et al. [19]. In that prospective randomized pilot trial, UDP irrigation was compared with conventional NaOCl irrigation in 28 patients over a 4-month follow-up period using the same PLAZEN RCT^®^ UDP device.

All procedures were performed under local anesthesia with 2% lidocaine containing 1:100,000 epinephrine and rubber dam isolation. After access cavity preparation, the working length was determined. A lip holder was placed at the corner of the patient’s mouth to serve as a return electrode and to complete the electrical circuit for real-time impedance feedback monitoring. The root canal was filled with physiological saline, and a K-file one to two sizes larger than the initial apical file was inserted to 3 mm short of the working length. Plasma discharge was activated by contacting the metal tip of the UDP device with the inserted K-file. The impedance feedback system continuously monitored electrical resistance between the device tip and the canal contents and automatically ceased energy delivery when impedance exceeded 5000 Ω [19].

**Figure 2:**
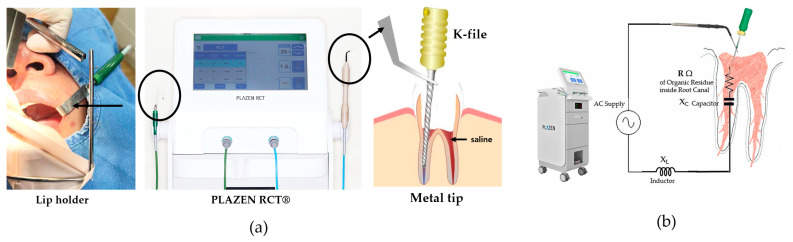
Clinical setup and equivalent circuit of the PLAZEN RCT^®^ underwater discharge plasma (UDP) system. (**a**) Clinical configuration showing the lip holder serving as a return electrode, the PLAZEN RCT^®^ UDP device, and the metal tip contacting a K-file inserted into a saline-filled root canal. (**b**) Equivalent RLC circuit illustrating the electrical pathway used for real-time impedance monitoring and impedance-based cutoff of energy delivery during UDP activation. The LCD display shown in panel (**a**) is part of the device interface and is not intended to present scientific data, experimental results, or study-specific parameter values. Created by the authors.

UDP application was performed in two sequential stages. The first discharge was applied after the working length was determined and before canal shaping. Canal shaping was then completed using nickel–titanium (NiTi) rotary instruments under continuous physiological saline irrigation. The second discharge was applied after canal shaping and immediately before obturation, using the same device configuration. In multirooted teeth, UDP activation was performed per canal; therefore, each canal received one activation at each stage. Thus, the total number of UDP activations per tooth depended on the number of canals, but the number of activations per canal was standardized. The clinical UDP application configuration and procedural sequence are illustrated in Figure 2.

The power setting for the UDP application was determined based on the preoperative PAI score, which served as a pragmatic radiographic surrogate for the presence and severity of periapical disease. Teeth with PAI scores of 1–2 were treated using 25 W for 1.5 s, whereas teeth with PAI scores of 3–5 were treated using 30 W for 1.5 s. These settings corresponded to energy delivery of 37.5 J and 45.0 J per activation, respectively. The operating frequency of the PLAZEN RCT^®^ UDP device was fixed at 510 kHz throughout all procedures, within the high-frequency range used for electrosurgical energy delivery. This frequency is consistent with that used in a previous study evaluating heat generation and temperature increase in the root during electromagnetic apical treatment [24].

Tooth anatomy, canal dimensions, and procedural complexity were not used as determinants of UDP power settings in the clinical protocol applied during the study period. Instead, procedural variability was addressed by standardized canal preparation, physiological saline irrigation, working length control, and per-canal UDP application in multirooted teeth. Although UDP activation may be less dependent on direct irrigant-wall contact than conventional chemical irrigation because plasma discharge occurs within a conductive aqueous medium, the extent to which canal anatomy influences discharge distribution has not been fully established. Therefore, the present parameter selection should be interpreted as a protocol derived from prior clinical application and device safety constraints, rather than as an independently validated anatomy- or complexity-specific dosing model. The appropriateness of anatomy- or complexity-adjusted UDP dosing should be evaluated in future prospective protocol-optimization studies.

For this study, UDP-related adverse events were specifically defined as thermal tissue injury caused by discharge heat, including heat-induced damage to the periodontal ligament or alveolar bone resulting from uncontrolled or excessive energy delivery during plasma activation. This definition reflects the principal device-specific risk of UDP: high-frequency electrical discharge in an aqueous medium could, in theory, cause thermal injury if energy delivery were uncontrolled. UDP-related adverse events were ascertained through systematic retrospective review of clinical records, operative notes, and serial periapical radiographs at each follow-up visit. General endodontic procedural complications, including canal transportation, ledging, perforation, and instrument separation, were assessed through review of operative notes and radiographic records and were distinguished from UDP-related adverse events unless temporally and mechanistically linked to plasma activation. Postoperative pain disproportionate to expected post-treatment discomfort and temporally associated with UDP activation, new or progressive periapical pathology on follow-up radiographs, or soft-tissue complications temporally linked to UDP use were recorded as potential device-related adverse events. No events meeting these criteria were identified in any of the 186 treated teeth over a maximum follow-up period of 30 months. However, because adverse-event ascertainment was based on retrospective record review, minor or undocumented events cannot be completely excluded.

### 2.4. Data Collection

The following data were collected for each included tooth: patient age at treatment, systemic medical history, tooth number, tooth type (anterior, premolar, or molar), arch (maxillary or mandibular), number of root canals (single or multiple canals), pulpal diagnosis, periapical diagnosis, retreatment status, periodontal probing depth > 3 mm, suspected crack, canal calcification, canal obliteration, preoperative pain, preoperative PAI score, number of treatment visits (single or multiple), date of obturation, follow-up dates and duration, presence of clinical signs or symptoms at follow-up, UDP-related adverse events, general endodontic procedural complications, follow-up periapical radiographs, and follow-up PAI scores. General endodontic procedural complications included canal transportation, ledging, perforation, and instrument separation, as documented in operative notes or radiographic records.

Although data were collected at the tooth level for the primary outcome analysis, patient-level baseline characteristics, including age distribution, systemic medical history, and the number of teeth contributed per patient, were summarized separately in Supplementary Table S5.

### 2.5. Radiographic Assessment

Periapical radiographs obtained preoperatively, immediately after obturation, and at the final follow-up visit were reviewed and assessed using the Periapical Index (PAI) scoring system (scores 1–5) proposed by Ørstavik et al. [25]. The analysis was conducted at the tooth level. For multi-rooted teeth, the highest (i.e., least favorable) PAI score among the roots was used to represent the entire tooth. Using the highest PAI score in multi-rooted teeth represents a conservative tooth-level approach that reflects the least favorable root-level periapical status. Although this method may classify some multi-rooted teeth as having more severe disease than would be suggested by an average or root-level summary, it reduces the risk of underestimating persistent periapical pathology in any affected root. In accordance with established criteria, teeth with a follow-up PAI score of 1 or 2 were classified as Healed, those with an improved but still elevated PAI score as Healing, and those without PAI improvement as Unhealed [26,27].

Radiographic evaluation was performed by two examiners, a board-certified oral and maxillofacial radiologist and an endodontist, both affiliated with departments external to the treating institution. Before the evaluation, both examiners completed a calibration session using 100 reference periapical radiographic images to standardize PAI scoring. Each examiner was provided with sets of three sequential radiographs per tooth, including preoperative, immediate post-obturation, and follow-up images. Therefore, the examiners were not blinded to image chronology. This limitation was considered unavoidable because assessment of periapical healing requires comparison between baseline and follow-up radiographs, and the treatment stage is visually apparent from the presence or absence of root canal filling material and coronal restoration. However, the examiners conducted the assessments independently and were blinded to all clinical information, treatment details, UDP application parameters, and predefined clinical success/failure classifications. Discrepancies between examiners were resolved by consensus. Inter-examiner reliability was quantified using the quadratic-weighted Cohen’s kappa coefficient [28].

To ensure independence in the adjudication of outcomes, all clinical records and sequential periapical radiographs were collected and compiled by a resident doctor in the Department of Conservative Dentistry who had not been involved in any of the treatment procedures. The compiled datasets were anonymized prior to submission to the external examiners. The treating clinician had no involvement in data collection, case compilation, or radiographic outcome assessment at any stage.

### 2.6. Treatment Outcome Assessment

Treatment success was defined as the simultaneous fulfillment of radiographic healing criteria and absence of clinical signs or symptoms. Clinical signs or symptoms included pain, sensitivity to percussion or palpation, swelling, or sinus tract formation. Overall success rates were calculated using Strict criteria (Healed + absence of clinical signs/symptoms) and Loose criteria (Healed or Healing + absence of clinical signs/symptoms) [22,29]. Stratified analysis was subsequently performed according to preoperative PAI score: Group A (PAI 1–2) and Group B (PAI 3–5). Because applying a uniform follow-up PAI threshold of ≤2 to Group A could overestimate success in teeth without definite preoperative periapical pathology, a distinct outcome criterion was applied to this group. For Group A, success required no PAI deterioration, no clinical signs or symptoms, and tooth survival [22,25]. Group B was evaluated using the Strict and Loose criteria based on Strindberg’s clinical framework [29]. Because the success criteria differed between Group A and Group B, their success rates should be interpreted independently and not directly compared. PAI-based group assignment was determined solely by the preoperative radiographic PAI score and therefore did not necessarily correspond to the clinical periapical diagnosis. For example, teeth clinically diagnosed with apical periodontitis, especially symptomatic apical periodontitis, may show minimal or no radiographic periapical changes in the early or acute phase and could therefore be classified as Group A (PAI 1–2).

All teeth meeting radiographic and/or clinical failure criteria were retained in the outcome analysis and classified as failures at the time point at which failure criteria were met, regardless of subsequent clinical management. In cases where treatment failure was identified during follow-up, the clinical response was nonsurgical root canal retreatment rather than extraction; no teeth were extracted due to endodontic treatment failure. These teeth were classified as failures in the primary outcome analysis irrespective of whether retreatment was subsequently initiated or completed.

### 2.7. Statistical Analysis

All statistical analyses were conducted with the tooth as the primary unit of analysis. The tooth was selected as the primary unit of analysis for the following reasons: periapical healing, as assessed by the PAI scoring system, is an inherently tooth-level outcome; tooth-level analysis is the conventional approach in the endodontic outcome literature and facilitates comparability with prior studies; and the majority of patients contributed only one tooth (101/134; 75.4%), limiting the extent of within-patient dependency. To account for potential within-patient clustering arising from patients contributing multiple teeth, cluster-aware sensitivity analyses were performed as described below.

Continuous variables are presented as mean ± standard deviation or median and range, depending on data distribution. Categorical variables are presented as frequencies and percentages. Between-group comparisons of continuous variables were performed using independent-samples *t*-tests or Mann–Whitney U tests, as appropriate. Categorical variables were compared using chi-squared tests or Fisher’s exact tests. Statistical significance was defined as a two-tailed *p* value < 0.05.

The primary objective of this study was to evaluate the periapical healing rate following physiological saline-based UDP irrigation. Prognostic factor analysis was conducted as an exploratory secondary analysis. Candidate variables were selected a priori based on clinical relevance and existing literature [23]. Based on the anticipated limited number of outcome events and the recommended events-per-variable (EPV) threshold of ≥10, the model was limited to three independent variables. The number of failures was 38 under Strict criteria and 33 under Loose criteria, yielding EPV values of 12.7 and 11.0, respectively. A multivariable logistic regression model was constructed using periodontal pocket depth > 3 mm, canal obliteration, and preoperative PAI score. Preoperative PAI score was modeled as an ordinal continuous variable, with 1-unit increments. Odds ratios (ORs) with 95% confidence intervals (CIs) were calculated. Because this analysis was exploratory, the findings were interpreted as hypothesis-generating only.

To assess the potential impact of attrition bias due to loss to follow-up, sensitivity analyses were conducted using three complementary approaches: best- and worst-case scenarios, tipping point analysis, and multiple imputation (MI; M = 20 datasets) conditioned on observed baseline covariates. MI was performed under the missing-at-random (MAR) assumption, whereby the probability of missing outcome data was modeled as depending on observed baseline covariates included in the imputation model but not on unobserved outcome values after conditioning on those covariates. The imputation model included baseline variables selected for clinical relevance and for observed imbalance between included and lost-to-follow-up teeth, including patient age, preoperative PAI score, number of root canals, periodontal pocket depth > 3 mm, retreatment status, pulpal diagnosis, and periapical diagnosis. Because the MAR assumption cannot be verified in a retrospective cohort, the MI results were interpreted as sensitivity estimates rather than definitive corrected outcome rates. Best-case, worst-case, and tipping point analyses were also performed to characterize the range of possible effects under alternative attrition scenarios. The same procedure was applied separately to Group B (PAI 3–5), which represented teeth with definite preoperative periapical pathology.

To account for potential within-patient clustering arising from the inclusion of multiple teeth per patient, the primary logistic regression model was additionally refitted using four cluster-aware approaches: cluster-robust standard errors, generalized estimating equation (GEE) models with exchangeable and first-order autoregressive working correlation structures, and a generalized linear mixed model (GLMM) with a patient-level random intercept. The intraclass correlation coefficient (ICC) was computed from the GLMM random intercept on the latent logit scale. All analyses were performed using R version 4.5.2 (R Foundation for Statistical Computing, Vienna, Austria).

## 3. Results

**Figure 3:**
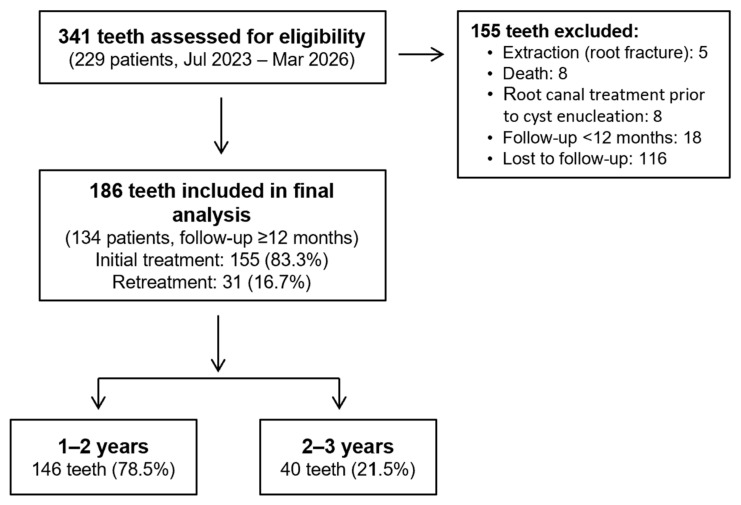
Flow diagram of tooth selection and follow-up in the retrospective cohort. The diagram shows the initially screened teeth, exclusion reasons, teeth lost to follow-up or with insufficient follow-up duration, and the final analytical cohort.

A total of 341 teeth from 229 patients were identified as potentially eligible. Of these, 155 teeth were excluded because of extraction due to root fracture (*n* = 5), death of the patient during the follow-up period (*n* = 8), root canal treatment performed prior to planned cyst enucleation (*n* = 8), follow-up duration of less than 12 months (*n* = 18), or refusal to attend follow-up or loss to follow-up (*n* = 116). The final analytical cohort comprised 186 teeth from 134 patients, corresponding to a tooth-level inclusion rate of 54.5% (186/341) and a patient-level inclusion rate of 58.5% (134/229). The selection and follow-up process is illustrated in Figure 3.

To assess potential attrition bias, baseline characteristics of the included teeth and teeth lost to follow-up were compared in Supplementary Table S1. Baseline characteristics differed significantly between the included teeth and teeth lost to follow-up in several variables, including age, tooth type, pulpal diagnosis, number of root canals, and preoperative PAI score. These findings indicate that loss to follow-up was not completely random and suggest the possibility of attrition-related selection bias.

Among the 186 included teeth, 146 teeth (78.5%) had a follow-up duration of 12 to <24 months, and 40 teeth (21.5%) had a follow-up duration of 24 to 30 months. The median patient age was 58 years (range, 14–89 years), and the median follow-up duration was 16 months (range, 12–30 months). Medians and ranges are reported as the primary descriptors for patient age and follow-up duration because both variables showed non-symmetric distributions. The mean patient age (53.7 years) was lower than the median because of younger outliers, whereas the mean follow-up duration (17.6 months) exceeded the median because of a small number of cases with longer follow-up.

**Figure 4:**
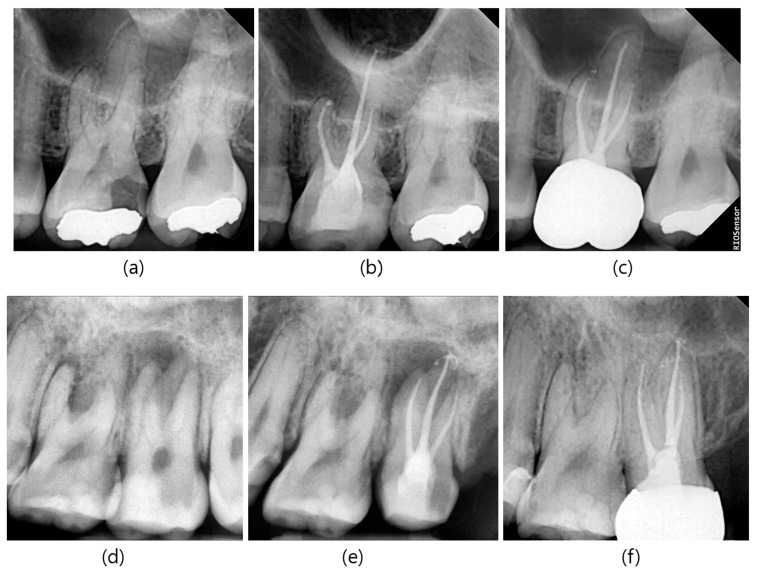
Representative serial periapical radiographs from Group A (preoperative PAI 1–2) and Group B (preoperative PAI 3–5). Images (**a**–**c**) show a Group A case, and images (**d**–**f**) show a Group B case at the preoperative stage (**a**,**d**), immediately after obturation (**b**,**e**), and final follow-up at ≥12 months after obturation (**c**,**f**). All radiographs were acquired using a standardized digital system (RIOS Sensor; Healdens, Yongin, Republic of Korea).

The tooth type distribution was as follows: molars, 84 teeth (45.2%); anterior teeth, 57 teeth (30.6%); and premolars, 45 teeth (24.2%). Initial RCT was performed in 155 teeth (83.3%), and retreatment was performed in 31 teeth (16.7%). Irreversible pulpitis was the most common pulpal diagnosis (*n* = 97; 52.2%). Detailed baseline characteristics are presented in Table 1. Based on preoperative PAI scores, 132 teeth (71.0%) were classified as Group A (PAI 1–2), and 54 teeth (29.0%) were classified as Group B (PAI 3–5). Representative serial periapical radiographs from each group are shown in Figure 4.

Inter-examiner reliability for radiographic assessment was substantial, with a quadratic-weighted Cohen’s kappa coefficient of 0.757 [28].

Radiographic healing outcomes and treatment success rates are summarized in Table 2. Among the 186 teeth, radiographic outcomes were classified as Healed (159; 85.5%), Healing (7; 3.8%), and Unhealed (20; 10.8%). Overall Strict and Loose success rates were 79.6% (148/186) and 82.3% (153/186), respectively. Initial RCT showed numerically higher success rates than retreatment under both the Strict criteria (81.3% vs. 71.0%) and the Loose criteria (83.2% vs. 77.4%). Detailed outcomes according to arch, tooth type, and treatment type are presented in Table 2.

Stratified outcomes according to preoperative PAI group are presented in Table 3. For Group A, treatment success was defined as no PAI deterioration, no clinical signs or symptoms, and tooth survival. In Group A (PAI 1–2; *n* = 132), the treatment success rate was 84.1% (111/132), with 100% tooth survival. Failure was associated with persistent clinical signs or symptoms in 13 teeth (9.8%) and PAI deterioration in 11 teeth (8.3%); these failure categories were not mutually exclusive. Among initially treated teeth in Group A, the success rate was 83.1% (103/124). Retreatment cases in Group A (*n* = 8) are presented in Table 3 for completeness only. The very small sample size precludes meaningful interpretation of the 100% success rate observed in this subgroup, and these data should not be generalized.

In Group B (PAI 3–5; *n* = 54), radiographic outcomes were classified as Healed in 38 teeth (70.4%), Healing in 7 teeth (13.0%), and Unhealed in 9 teeth (16.7%). Strict and Loose success rates were 68.5% (37/54) and 83.3% (45/54), respectively. Initial RCT showed numerically higher success rates than retreatment under both the Strict criteria (74.2% vs. 60.9%) and the Loose criteria (87.1% vs. 78.3%). Because different success criteria were applied to Group A and Group B, the outcomes of these groups should be interpreted independently and not directly compared.

The results of the exploratory prognostic factor analysis are presented in Table 4. In the multivariable logistic regression model applied to all 186 teeth, periodontal pocket depth > 3 mm was significantly associated with lower odds of treatment success under both the Strict and Loose criteria (Strict: OR, 0.24; 95% CI, 0.10–0.54; *p* < 0.001; Loose: OR, 0.25; 95% CI, 0.11–0.59; *p* = 0.001). Canal obliteration was associated with lower odds of success under the Loose criteria in the primary model (OR, 0.23; 95% CI, 0.06–0.97; *p* = 0.046) and showed a non-significant trend under the Strict criteria (OR, 0.26; 95% CI, 0.06–1.09; *p* = 0.065). Preoperative PAI score, modeled per 1-unit increment, showed a marginal association with lower odds of success under the Strict criteria (OR, 0.76; 95% CI, 0.58–1.00; *p* = 0.050) but was not associated with success under the Loose criteria (OR, 0.99; 95% CI, 0.73–1.34; *p* = 0.958). Because this analysis was exploratory, these findings should be interpreted as hypothesis-generating only. In particular, borderline associations, including canal obliteration under the Loose criteria and preoperative PAI score under the Strict criteria, should be interpreted with caution, as these estimates were less consistent in cluster-aware sensitivity analyses and should not be interpreted as established prognostic factors.

Sensitivity analyses were performed to assess the potential impact of attrition bias. Best- and worst-case analyses were performed by assuming that all 116 teeth lost to follow-up were classified as treatment successes or failures, respectively. A tipping point analysis was performed to determine the minimum proportion of lost-to-follow-up teeth that would need to have failed for the observed success rate to fall below the Strict success estimate reported by Burns et al. [2]. Multiple imputation (MI; M = 20 datasets) was performed using logistic regression conditioned on observed baseline covariates, including age, preoperative PAI score, number of root canals, periodontal pocket depth, retreatment status, and preoperative periapical diagnosis; pooled estimates were derived according to Rubin’s rules. The MI-adjusted Strict success rate was 82.2% (95% CI, 77.0–87.3%); however, this estimate relies on the MAR assumption and should be interpreted as a sensitivity estimate rather than a corrected outcome rate. The tipping point analysis indicated that 53.2% of the 116 lost-to-follow-up teeth (*n* = 62) would have needed to fail for the Strict success rate to fall below the reference threshold. The worst-case scenario yielded a substantially lower Strict success rate of 49.0%, indicating that the primary estimates remain sensitive to assumptions about missing outcomes. For Group B (PAI 3–5), tipping point thresholds were referenced against the Strict success rates reported by Sunde et al. [30] and Artaza et al. [31]; the tipping point was 20.1% (*n* = 3). Results of the attrition sensitivity analyses are presented in Supplementary Figure S1.

Cluster-aware sensitivity analyses were performed to assess the potential influence of within-patient clustering and are presented in Supplementary Tables S3 and S4. Of the 134 patients, 101 (75.4%) contributed one tooth, 18 (13.4%) contributed two teeth, 11 (8.2%) contributed three teeth, and 4 (3.0%) contributed four teeth (mean teeth per patient: 1.39 [SD 0.76]; full cluster size distribution is presented in Supplementary Table S2). The majority of patients contributed only a single tooth, indicating that within-patient clustering was present but limited in extent. In the Strict success model, the GLMM-derived ICC was 0.232, indicating moderate within-patient correlation. Periodontal pocket depth > 3 mm remained consistently associated with lower odds of Strict success across cluster-aware models, whereas associations involving canal obliteration and preoperative PAI score were less consistent. In the Loose success model, the GLMM-derived ICC was high and was accompanied by very wide confidence intervals for the GLMM estimates, suggesting model instability due to the limited number of multi-tooth clusters and the sparse data structure for random-effects estimation. Therefore, the GLMM results for Loose success should be interpreted with caution, and greater emphasis should be placed on the cluster-robust and GEE sensitivity analyses. Overall, the cluster-aware analyses supported the consistency of the association between periodontal pocket depth > 3 mm and lower treatment success, while indicating that canal obliteration and preoperative PAI score should not be interpreted as established prognostic factors.

## 4. Discussion

This retrospective cohort study evaluated short- to medium-term clinical and radiographic outcomes after nonsurgical RCT using physiological saline-based UDP irrigation in a heterogeneous real-world clinical cohort. Overall Strict and Loose success rates were 79.6% and 82.3%, respectively, and initial RCT showed numerically higher success rates than retreatment under both the Strict criteria (81.3% vs. 71.0%) and the Loose criteria (83.2% vs. 77.4%). No UDP-related adverse events were recorded during follow-up. These findings provide preliminary descriptive evidence supporting the clinical feasibility of physiological saline-based UDP irrigation. Because no concurrent control group was included, no inference regarding comparative efficacy relative to NaOCl-based irrigation can be drawn from these data [2,32,33]. The present study evaluated a NaOCl-free protocol in which physiological saline irrigation was combined with UDP activation, while other major components of the root canal treatment procedure, including canal preparation, obturation materials, and coronal restoration, were standardized. The observed outcomes should therefore be interpreted as descriptive clinical outcomes of this specific protocol and should not be considered evidence of equivalence, superiority, or non-inferiority compared with conventional NaOCl-based endodontic protocols.

A key contribution of this study is that it builds on the early clinical feasibility data reported in the preceding prospective randomized pilot trial by Lyu et al. [19] by evaluating a larger real-world observational cohort with longer follow-up. That pilot trial compared UDP-based irrigation with conventional NaOCl irrigation and reported short-term clinical feasibility with no device-related adverse events; however, its sample size of 28 patients and 4-month follow-up period were insufficient to characterize medium-term periapical healing. The present study addresses this gap by evaluating clinical and radiographic outcomes in 186 teeth with a minimum follow-up of 12 months and a maximum follow-up of 30 months.

The methodological differences between the two studies should also be considered. The pilot trial had the strengths of randomized allocation and a concurrent control group but was limited by its short follow-up period and small sample size, which precluded meaningful prognostic factor analysis. In contrast, the present study used a retrospective single-arm design but provides complementary observational data from a larger cohort with longer follow-up and heterogeneous clinical cases, including multi-rooted teeth and retreatment cases.

A further methodological distinction of the present study is the use of stratified success criteria based on preoperative periapical status. Conventional root canal outcome studies have often applied a uniform follow-up PAI threshold of ≤2 to define success. However, as noted by Liu et al., including teeth without preoperative periapical pathology in aggregate analyses may overestimate success rates [34]. Similarly, Sunde et al. excluded PAI 1–2 cases from their cohort to mitigate this bias [30]. In the present study, teeth were stratified into Group A (PAI 1–2) and Group B (PAI 3–5), and distinct success criteria were applied to each group to reduce the risk of outcome inflation. For Group A, success required no PAI deterioration, no clinical signs or symptoms, and tooth survival. For Group B, the Strict and Loose criteria based on Strindberg’s clinical framework were applied [29]. Because the criteria differed between the two groups, their success rates should be interpreted independently and not directly compared.

Using these group-specific criteria, Group A showed a success rate of 84.1% with 100% tooth survival, whereas Group B showed success rates of 68.5% and 83.3% under the Strict and Loose criteria, respectively. It should be noted that many published endodontic outcome studies include teeth across the full preoperative PAI spectrum in their primary analyses; therefore, the inclusion of Group A in the present study was intended to maintain comparability with this literature. However, because the teeth in Group A lacked definite preoperative periapical pathology, high success and survival rates are expected in this group regardless of the irrigant or activation protocol used. Accordingly, Group A outcomes should not be overinterpreted as evidence of enhanced therapeutic effectiveness of UDP-based irrigation. The stratified reporting approach was used to contextualize the overall cohort outcomes while reducing the risk of outcome inflation attributable to radiographically uninvolved teeth. Outcomes in Group B are more clinically informative for evaluating periapical healing after established periapical disease, whereas Group A outcomes should primarily be interpreted as maintenance-related outcomes.

To provide clinical context for the observed outcomes, selected NaOCl-based endodontic outcome studies were identified according to predefined criteria: PAI-based success criteria, a minimum follow-up of 12 months, explicit reporting of both Strict and Loose success rates, sample size ≥ 30, and stratified analysis by treatment type or preoperative PAI score [2,30,31,32,33,34,35,36,37]. Across these studies, reported Strict success rates ranged from 67% to 82%, and reported Loose success rates ranged from 83% to 93%. For teeth with definite preoperative periapical pathology (PAI 3–5), Strict success rates of 67–71% have been reported in cohort studies using comparable PAI-based outcome definitions [30,33,34]. These values are presented only to describe the broader reference landscape of published endodontic outcome studies and should not be interpreted as a comparative benchmark for the present cohort. Because the present study lacked a concurrent NaOCl control group, it cannot determine whether physiological saline-based UDP irrigation is equivalent, superior, or inferior to conventional NaOCl-based irrigation. Accordingly, no comparative or non-inferiority inference should be drawn from these descriptive findings. The published literature is referenced solely to provide methodological context at this early stage of UDP clinical evidence development [2,32,33].

In Group A, PAI deterioration occurred in 11 teeth (8.3%), and new clinical signs or symptoms developed in 13 teeth (9.8%), yielding an overall failure rate of 15.9% (21/132). These failure categories were not mutually exclusive. Such unfavorable outcomes may not be attributable solely to inadequate canal disinfection, given the potential contribution of non-intracanal or non-endodontic factors, including coronal microleakage due to restoration failure, inadequate marginal sealing, periodontal status, and periodontal ligament responses secondary to occlusal overload. Future studies should incorporate restorative, occlusal, and periodontal prognostic variables to better characterize the determinants of post-treatment outcomes.

In Group B, the combined radiographic improvement rate (Healed + Healing) was 83.4%, whereas 70.4% of teeth were classified as Healed. This finding suggests that complete radiographic resolution of preoperative periapical pathology may not occur within a short- to medium-term follow-up period. This interpretation is consistent with the established role of preoperative periapical status as a negative prognostic factor, as reported by Burns et al. [2]. The seven teeth classified as Healing (13.0%) at the median follow-up of 16 months may progress to Healed with longer observation [20].

UDP is a subtype of non-thermal plasma (NTP), which is characterized by the generation of reactive species under conditions in which bulk thermal elevation of the surrounding medium is limited. This property provides a theoretical basis for reducing the risk of heat-induced tissue injury compared with thermal energy-based approaches; however, device-specific safety must be evaluated clinically. Previous literature on non-thermal plasma applications in endodontics and dentistry, as well as studies evaluating root surface temperature changes during endodontic procedures, provides a general context for thermal safety assessment but does not establish the device-specific safety of UDP-based root canal irrigation [38,39]. In the present cohort, no UDP-related adverse events, defined as thermal tissue injury caused by discharge heat, were recorded among 186 treated teeth over a maximum follow-up period of 30 months. This finding is consistent with the absence of device-related adverse events reported in the predecessor pilot trial by Lyu et al. [19]. However, because adverse-event ascertainment was based on retrospective review of clinical records, operative notes, and serial periapical radiographs, minor or undocumented events cannot be completely excluded. Therefore, the present findings should be interpreted as preliminary observational safety data rather than definitive evidence of clinical safety.

The stability of the primary outcome estimates was evaluated through sensitivity analyses addressing attrition bias and within-patient clustering. Multiple imputation yielded an adjusted Strict success estimate similar to the observed estimate; however, this result relies on the missing-at-random assumption and should be interpreted as a sensitivity estimate rather than a corrected outcome rate. The worst-case analysis yielded a substantially lower Strict success rate of 49.0%, indicating that the primary outcome estimates remain sensitive to assumptions regarding missing outcomes. Cluster-aware analyses showed that periodontal pocket depth > 3 mm remained the most consistent factor associated with lower treatment success, whereas associations involving canal obliteration and preoperative PAI score were less stable. These findings reinforce the need to interpret the primary outcome estimates, subgroup analyses, and prognostic factor analyses cautiously.

Because all treatments were performed by a single experienced clinician in a standardized clinical setting, the present findings may primarily reflect outcomes observed under operator- and protocol-specific conditions. Although the PLAZEN RCT^®^ UDP device has been introduced into clinical practice, the generalizability of the present findings, particularly with respect to reproducibility across operators with different levels of experience and across multicenter clinical environments, requires independent validation. Future multicenter, multi-operator randomized controlled trials are needed to evaluate the reproducibility, comparative effectiveness, and long-term outcomes of physiological saline-based UDP irrigation.

This study has several limitations that warrant consideration. The single-center retrospective design introduces the possibility of selection and information bias. In addition, no a priori sample size or precision calculation was performed; the cohort size was determined pragmatically by including all consecutive eligible teeth treated during the study period. Accordingly, the present study was not powered to detect a prespecified effect size, and the available sample size limits the precision of the outcome estimates. The absence of a concurrent control group also limits direct comparison between UDP and conventional NaOCl-based irrigation. Although the outcomes of the present cohort were contextualized against published NaOCl-based reference data [2,32,33], these comparisons should not be interpreted as a direct head-to-head comparison or a meta-analytic synthesis. A prospective randomized controlled trial with a concurrent NaOCl control arm and formal sample size calculation is required to draw definitive comparative conclusions.

Several sources of residual confounding should also be considered. Potentially relevant prognostic variables, such as canal preparation taper, quality of coronal restoration, occlusal stability, and other restorative or periodontal factors, were not systematically collected, limiting the ability to fully control for residual confounding. In addition, the median follow-up period of 16 months may be insufficient for evaluating long-term healing trajectories, as complete radiographic resolution may require several years [20,26].

Loss to follow-up was substantial. Although baseline comparisons between the included cohort and teeth lost to follow-up were performed (Supplementary Table S1), the observed baseline differences suggest that loss to follow-up was not completely random. Prespecified attrition sensitivity analyses, including best-case/worst-case scenarios, tipping point analysis, and multiple imputation, were conducted to evaluate the potential impact of attrition (Supplementary Figure S1). Multiple imputation yielded an adjusted Strict success estimate of 82.2%, which was similar to the observed-case estimate; however, this estimate relies on the missing-at-random assumption, which cannot be verified in this retrospective cohort. Because baseline characteristics differed between included and lost-to-follow-up teeth, informative censoring and missing-not-at-random mechanisms cannot be excluded. The worst-case Strict success rate of 49.0%, derived by classifying all 116 teeth lost to follow-up as treatment failures, should therefore be regarded as an important lower-bound estimate for interpreting the uncertainty introduced by attrition. Accordingly, the primary outcome estimates should be interpreted with caution, and future studies should incorporate active recall systems and prespecified MNAR sensitivity analyses.

No microbiological assessment was performed in the present study; therefore, the antimicrobial efficacy of UDP-based irrigation in vivo cannot be established from the present data. The mechanistic rationale for UDP, including RONS generation, biofilm disruption, and broad-spectrum antimicrobial activity, is supported by preclinical and in vitro evidence only [11,12,13,14,15]. Radiographic periapical healing reflects a composite outcome influenced by multiple biological processes beyond intracanal disinfection alone and does not constitute direct evidence of antimicrobial efficacy. Furthermore, radiographic healing does not necessarily indicate complete histological healing [40].

In addition, all treatments were performed by a single experienced clinician; therefore, reproducibility across operators with different levels of experience and across multicenter settings requires further validation. The UDP application parameters used in this study were derived from the predecessor pilot trial [19] and have not been independently validated in separate clinical studies. As UDP-based endodontic irrigation is an emerging modality with limited clinical evidence, future studies should systematically evaluate parameter optimization, including power level, discharge duration, number of activations, and the influence of canal anatomy and disease severity.

Complete blinding of radiographic assessors to image chronology was not achievable because sequential periapical radiographs contain inherent visual cues indicating the treatment stage. The preoperative radiograph shows untreated root canal anatomy without obturation material, the immediate post-obturation radiograph shows root filling material within the canal space, and the follow-up radiograph often shows definitive coronal restoration. This limitation is common in endodontic outcome research using serial periapical radiographs. To minimize the risk of assessment bias within these constraints, radiographic evaluation was performed by two independent external examiners blinded to all clinical information and treatment details; a calibration session was conducted prior to evaluation, and discrepancies were resolved by consensus. Although the primary analysis was conducted at the tooth level and multiple teeth from the same patient were included in some cases, the potential influence of within-patient clustering was evaluated through prespecified clustering sensitivity analyses using cluster-robust standard errors, GEE, and GLMM approaches (Supplementary Tables S2–S4). These analyses helped assess the influence of clustering, but they should be regarded as supportive rather than definitive, given the limited number of clustered observations. Associations involving canal obliteration and preoperative PAI score were less consistent across cluster-aware models and should therefore be interpreted cautiously.

Finally, radiographic outcome assessment relied on two-dimensional periapical radiography with conventional PAI scoring. Although a CBCT-based periapical index offers higher sensitivity for periapical lesion detection and volumetric assessment [41], routine CBCT acquisition was not feasible in this retrospective real-world cohort. In addition, two-dimensional periapical radiography remains a widely used outcome measure in endodontic outcome research and allows comparability with the existing literature. Future prospective trials should consider incorporating CBCT-based assessment to more accurately evaluate residual lesions and healing dynamics.

## 5. Conclusions

This retrospective cohort study provides preliminary descriptive evidence on the clinical and radiographic outcomes observed after nonsurgical RCT using physiological saline-based UDP irrigation without NaOCl in a single-center, real-world cohort. The overall Strict and Loose success rates were 79.6% and 82.3%, respectively, and no UDP-related adverse events were recorded over a maximum follow-up period of 30 months. However, loss to follow-up was substantial, and the worst-case Strict success rate decreased to 49.0% when all 116 teeth lost to follow-up were classified as treatment failures. Therefore, these findings should be interpreted as descriptive outcome estimates for this specific physiological saline-based UDP protocol and do not constitute evidence of comparative efficacy or definitive clinical safety relative to conventional NaOCl-based irrigation. Stratified success criteria based on preoperative PAI score may help reduce outcome inflation in mixed endodontic cohorts and may serve as a useful methodological framework for future studies. Adequately powered prospective randomized controlled trials with concurrent NaOCl control arms and long-term follow-up are needed to evaluate the comparative effectiveness, safety, and reproducibility of physiological saline-based UDP irrigation protocols.

## Figures and Tables

**Table 1:** Baseline clinical and radiographic characteristics of the included teeth (*n* = 186).

Variables	Total (*n* = 186)
Patient demographics	
Age, median (range) (years)	58 (14–89)
Follow-up period, median (range) (months)	16 (12–30)
Preoperative PAI score, mean ± SD	2.1 ± 1.3
Tooth distribution, *n* (%)	
Anterior	57 (30.6)
Premolar	45 (24.2)
Molar	84 (45.2)
Arch, *n* (%)	
Maxilla	112 (60.2)
Mandible	74 (39.8)
Number of root canals, *n* (%)	
Single canal	78 (41.9)
Multiple canals	108 (58.1)
Treatment type, *n* (%)	
Initial treatment	155 (83.3)
Retreatment	31 (16.7)
Number of visits, *n* (%)	
Single visit	103 (55.4)
Multiple visits	83 (44.6)
Clinical findings, *n* (%)	
Periodontal pocket depth > 3 mm	35 (18.8)
Crack suspected	30 (16.1)
Pulpal diagnosis, *n* (%)	
Irreversible pulpitis	97 (52.2)
Necrotic pulp	54 (29.0)
Previously treated	31 (16.7)
Previously initiated therapy	4 (2.2)
Periapical diagnosis, *n* (%)	
Normal apical tissues	114 (61.3)
Symptomatic apical periodontitis	26 (14.0)
Asymptomatic apical periodontitis	24 (12.9)
Chronic apical abscess	22 (11.8)

Values are presented at the tooth level unless otherwise specified. Patient age is reported at the time of treatment. PAI = Periapical Index; SD = Standard Deviation. Four teeth with a history of pulpotomy only were classified as previously initiated therapy and as initial treatment cases because the root canal system had not been previously instrumented or obturated. Thus, “previously treated” corresponded to retreatment cases involving previously obturated canals.

**Table 2:** Radiographic healing status and Strict and Loose treatment success rates in the overall cohort and clinical subgroups.

	Radiographic Healing, *n* (%)			Treatment Success, *n* (%)		*p* Value ^†^	
	Healed	Healing	Unhealed	Strict	Loose	Strict	Loose
All teeth (*n* = 186)	159 (85.5)	7 (3.8)	20 (10.8)	148 (79.6)	153 (82.3)	—	—
By jaw location							
Maxillary (*n* = 112)	100 (89.3)	1 (0.9)	11 (9.8)	92 (82.1)	93 (83.0)	0.353	0.845
Mandibular (*n* = 74)	59 (79.7)	6 (8.1)	9 (12.2)	56 (75.7)	60 (81.1)		
By tooth type—Maxillary (3-group comparison)							
Anterior (*n* = 35)	33 (94.3)	0 (0.0)	2 (5.7)	30 (85.7)	30 (85.7)	0.720	0.690
Premolar (*n* = 27)	21 (77.8)	0 (0.0)	6 (22.2)	21 (77.8)	21 (77.8)		
Molar (*n* = 50)	46 (92.0)	1 (2.0)	3 (6.0)	41 (82.0)	42 (84.0)		
By tooth type—Mandibular (3-group comparison)							
Anterior (*n* = 22)	14 (63.6)	4 (18.2)	4 (18.2)	14 (63.6)	17 (77.3)	0.072	0.250
Premolar (*n* = 18)	17 (94.4)	0 (0.0)	1 (5.6)	17 (94.4)	17 (94.4)		
Molar (*n* = 34)	28 (82.4)	2 (5.9)	4 (11.8)	25 (73.5)	26 (76.5)		
By treatment type							
Initial treatment (*n* = 155)	136 (87.7)	4 (2.6)	15 (9.7)	126 (81.3)	129 (83.2)	0.223	0.445
Retreatment (*n* = 31)	23 (74.2)	3 (9.7)	5 (16.1)	22 (71.0)	24 (77.4)		

Healed: follow-up PAI ≤ 2; Healing: follow-up PAI improved but remained ≥3; Unhealed: follow-up PAI not improved. Strict = Healed + absence of clinical signs/symptoms; Loose = Healed or Healing + absence of clinical signs/symptoms. Clinical signs/symptoms at follow-up include pain, sensitivity to percussion or palpation, swelling, or sinus tract formation, according to Strindberg’s criteria [29]. ^†^ *p* values were calculated using Fisher’s exact test for two-group comparisons, including Maxillary vs. Mandibular and Initial treatment vs. Retreatment, and Pearson’s chi-square test for three-group comparisons of tooth type within Maxillary and Mandibular. These subgroup comparisons were exploratory and were not adjusted for multiple testing; therefore, *p* values are provided to aid interpretation of numerical differences and should not be interpreted as confirmatory evidence of subgroup effects. PAI = Periapical Index.

**Table 3:** Treatment outcomes according to preoperative PAI group and group-specific success criteria.

**Group A (PAI 1–2, *n* = 132)**	**Treatment Outcome, *n* (%)**				**Reason for Failure, *n* (%)**	
	**Success**		**Failure**		**Symptom Persistence**	**PAI Deterioration**
All teeth (*n* = 132)	111 (84.1)		21 (15.9)		13 (9.8)	11 (8.3)
By treatment type						
Initial treatment (*n* = 124)	103 (83.1)		21 (16.9)		13 (10.5)	11 (8.9)
Retreatment (*n* = 8)	8 (100.0)		0 (0.0)		—	—
**Group B (PAI 3–5, *n* = 54)**	**Radiographic Healing, *n* (%)**				**Strict Success, *n* (%)**	**Loose Success, *n* (%)**
	**Healed**	**Healing**		**Unhealed**	***n* (%)**	***n* (%)**
All teeth (*n* = 54)	38 (70.4)	7 (13.0)		9 (16.7)	37 (68.5)	45 (83.3)
By treatment type						
Initial treatment (*n* = 31)	23 (74.2)	4 (12.9)		4 (12.9)	23 (74.2)	27 (87.1)
Retreatment (*n* = 23)	15 (65.2)	3 (13.0)		5 (21.7)	14 (60.9)	18 (78.3)

Group A success criterion: no PAI deterioration, no clinical signs or symptoms, and tooth survival. Failure categories in Group A are not mutually exclusive. Group B criteria: Strict = Healed (PAI ≤ 2) + absence of clinical signs/symptoms; Loose = Healed or Healing + absence of clinical signs/symptoms. Success rates between Group A and Group B should not be directly compared because different criteria were applied. PAI = Periapical Index.

**Table 4:** Exploratory multivariable logistic regression analysis of candidate factors associated with treatment success under Strict and Loose criteria (*n* = 186).

Variable	Strict Criteria			Loose Criteria		
	OR	95% CI	*p*	OR	95% CI	*p*
Periodontal pocket depth > 3 mm vs. ≤3 mm	0.24	0.10–0.54	<0.001 *	0.25	0.11–0.59	0.001 *
Canal obliteration present vs. absent	0.26	0.06–1.09	0.065	0.23	0.06–0.97	0.046 *
Preoperative PAI (per 1-unit increase)	0.76	0.58–1.00	0.050	0.99	0.73–1.34	0.958

OR = odds ratio; CI = confidence interval; PAI = Periapical Index. All variables were entered simultaneously in the prespecified model. Strict criteria: Healed + absence of clinical signs/symptoms. Loose criteria: Healed or Healing + absence of clinical signs/symptoms. These analyses were exploratory and should be interpreted as hypothesis-generating. * *p* < 0.05.

## Data Availability

The data presented in this study are available on reasonable request from the corresponding author.

## Supplementary Materials

The following supporting information can be downloaded at https://www.mdpi.com/article/10.3390/biomedicines14061389/s1, Figure S1: Attrition bias sensitivity analysis for the overall cohort (A) and Group B (PAI 3–5) (B). Each panel presents four estimates: worst-case scenario, in which all teeth lost to follow-up were classified as failures; observed estimate, based on the included cohort only; multiple imputation (MI)-adjusted estimate, derived from logistic regression conditioned on selected baseline covariates (M = 20 datasets, Rubin’s rules); and best-case scenario, in which all teeth lost to follow-up were classified as successes. Error bars for the observed and MI-adjusted estimates represent 95% confidence intervals. In panel (A), the shaded band and dashed line indicate the 95% CI and pooled Strict success estimate reported by Burns et al. [2] for primary root canal treatment in conventional NaOCl-based endodontic outcome studies. In panel (B), the shaded band and reference lines indicate the Strict success rate range reported by Sunde et al. [30] and Artaza et al. [31] for cohorts restricted to teeth with preoperative periapical pathology. These reference estimates are provided for contextual interpretation only and do not represent a direct comparison between UDP- and NaOCl-based irrigation protocols. Tipping point values are indicated within each panel. MI, multiple imputation; NaOCl, sodium hypochlorite; PAI, Periapical Index; Table S1: Comparison of baseline characteristics between included teeth and teeth lost to follow-up; Table S2: Patient and tooth cluster description for within-patient clustering sensitivity analysis; Table S3: Sensitivity analysis accounting for within-patient clustering—Strict success; Table S4: Sensitivity analysis accounting for within-patient clustering—Loose success; Table S5: Patient-level baseline characteristics of the analytical cohort (*n* = 134 patients).
