# Supplementary material for: Clinical Outcomes of Plasma-Assisted Saline Irrigation in Nonsurgical Root Canal Treatment: A Preliminary Retrospective Cohort Study
Source: Biomedicines. 2026 Jun 19;14(6):1389. doi: 10.3390/biomedicines14061389 (PMC13296406; doi:10.3390/biomedicines14061389)
Supplement: Supplementary file 1 [file biomedicines-14-01389-s001.zip › biomedicines-4340269-supplementary.pdf]

Supplementary Table S1. Comparison of baseline characteristics between included teeth and teeth lost to follow-up.

Variable	Included Cohort (n = 186)	Lost to Follow-up (n = 116)	p-value	Standardized Mean Difference (SMD)
**Age (years), median (IQR)**	58 (41–68)	64 (55–74)	<0.001	0.423
**Tooth type, n (%)**			0.014	
Anterior	57 (30.6)	48 (41.4)		0.225
Premolar	45 (24.2)	35 (30.2)		0.135
Molar	84 (45.2)	33 (28.4)		0.343
**Arch, n (%)**			0.339	
Maxilla	112 (60.2)	63 (54.3)		0.119
Mandible	74 (39.8)	53 (45.7)		0.119
**Root canals, n (%)**			<0.001	
Single-rooted	78 (41.9)	73 (62.9)		0.420
Multi-rooted	108 (58.1)	43 (37.1)		0.420
**Pulpal diagnosis, n (%)**			<0.001	
Irreversible Pulpitis	97 (52.2)	88 (75.9)		0.487
Necrotic Pulp	54 (29.0)	12 (10.3)		0.484
Previously Treated	31 (16.7)	16 (13.8)		0.134
Previously initiated therapy	4 (2.2)	0 (0.0)		0.210
**Periapical diagnosis, n (%)**			0.004	
Normal Apical Tissues	114 (61.3)	90 (77.6)		0.348
Symptomatic AP	26 (14.0)	13 (11.2)		0.083
Asymptomatic AP	24 (12.9)	11 (9.5)		0.083
Chronic Apical Abscess	22 (11.8)	2 (1.7)		0.374
**Preoperative PAI score, mean ± SD**	2.1 ± 1.3	1.4 ± 0.7	<0.001	0.630
**Retreatment, n (%)**	31 (16.7)	16 (13.8)	0.625	0.079
**Periodontal pocket depth >3 mm, n (%)**	35 (18.8)	18 (15.5)	0.535	0.087
**Number of visits, n (%)**			0.23	
Single visit	103 (55.4)	73 (62.9)		0.150
Multiple visits	83 (44.6)	43 (37.1)		0.150

Data are presented as median (IQR) for age and mean ± SD or n (%) for other variables. Age was compared using the Mann-Whitney U test; all other continuous variables were compared using independent t-tests; categorical variables were compared using Fisher's exact test or χ^2 test. AP = Apical Periodontitis; IQR = Interquartile Range; PAI = Periapical Index; LTFU = Lost to Follow-up.

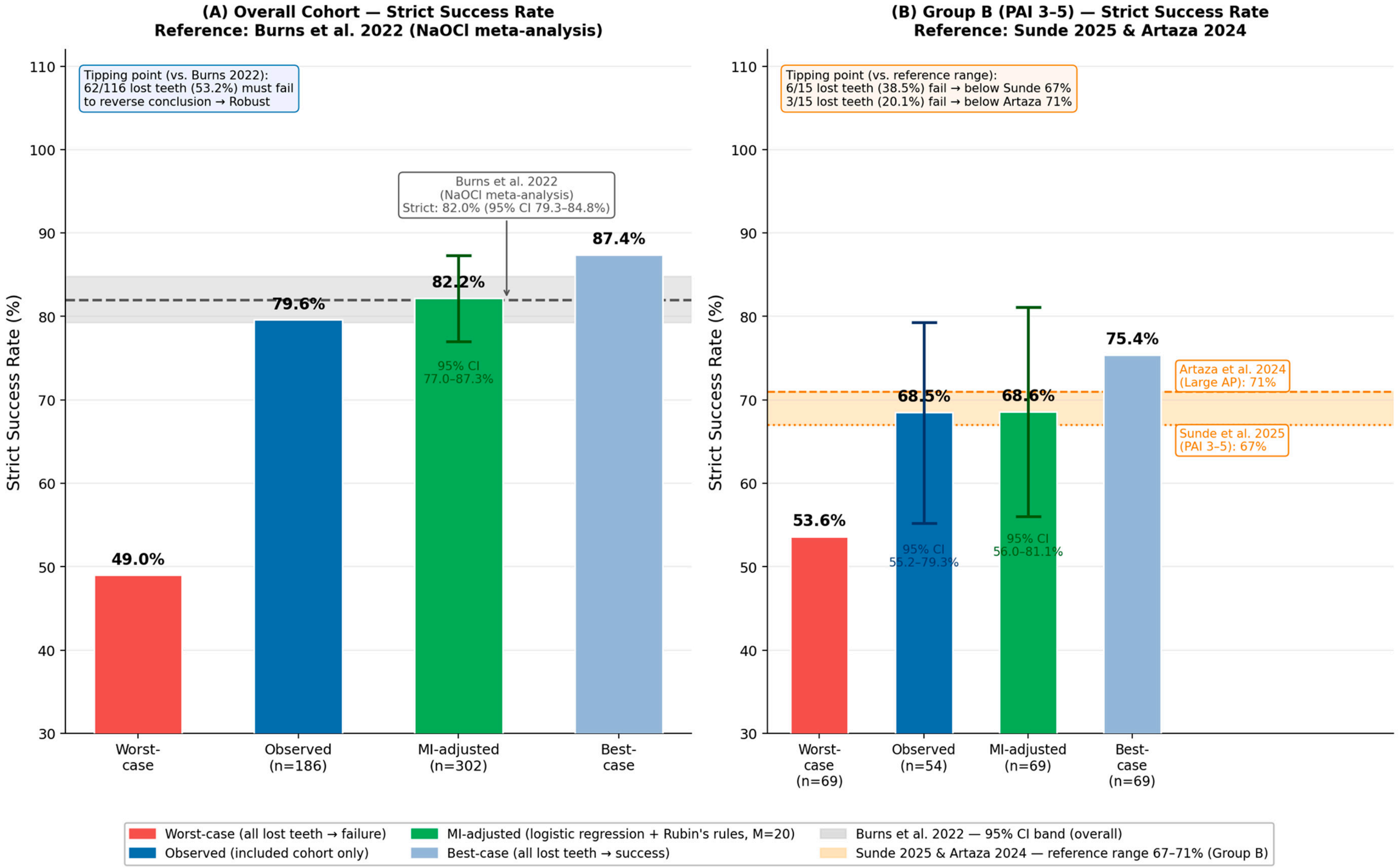

(A) Overall Cohort — Strict Success Rate
Reference: Burns et al. 2022 (NaOCl meta-analysis)
Tipping point (vs. Burns 2022):
62/116 lost teeth (53.2%) must fail
to reverse conclusion → Robust
Burns et al. 2022
(NaOCl meta-analysis)
Strict: 82.0% (95% CI 79.3–84.8%)
Strict Success Rate (%)
49.0%
79.6%
82.2%
87.4%
95% CI
77.0–87.3%
Worst-case
Observed (n=186)
MI-adjusted (n=302)
Best-case
(B) Group B (PAI 3–5) — Strict Success Rate
Reference: Sunde 2025 & Artaza 2024
Tipping point (vs. reference range):
6/15 lost teeth (38.5%) fail → below Sunde 67%
3/15 lost teeth (20.1%) fail → below Artaza 71%
Artaza et al. 2024
(Large AP): 71%
Sunde et al. 2025
(PAI 3–5): 67%
53.6%
68.5%
68.6%
75.4%
95% CI
55.2–79.3%
95% CI
56.0–81.1%
Worst-case (n=69)
Observed (n=54)
MI-adjusted (n=69)
Best-case (n=69)
Worst-case (all lost teeth → failure)
Observed (included cohort only)
MI-adjusted (logistic regression + Rubin's rules, M=20)
Best-case (all lost teeth → success)
Burns et al. 2022 — 95% CI band (overall)
Sunde 2025 & Artaza 2024 — reference range 67–71% (Group B)

Supplementary Figure S1. Attrition bias sensitivity analysis for the overall cohort (A) and Group B (PAI 3–5) (B).

Each panel presents four estimates: worst-case scenario, in which all teeth lost to follow-up were classified as failures; observed estimate, based on the included cohort only; multiple imputation (MI)-adjusted estimate, derived from logistic regression conditioned on selected baseline covariates (M = 20 datasets, Rubin's rules); and best-case scenario, in which all teeth lost to follow-up were classified as successes. Error bars for the observed and MI-adjusted estimates represent 95% confidence intervals. In panel (A), the shaded band and dashed line indicate the 95% CI and pooled Strict success estimate reported by Burns et al. [2] for primary root canal treatment in conventional NaOCl-based endodontic outcome studies. In panel (B), the shaded band and reference lines indicate the Strict success rate range reported by Sunde et al. [30] and Artaza et al. [31] for cohorts restricted to teeth with preoperative periapical pathology. These reference estimates are provided for contextual interpretation only and do not represent a direct comparison between UDP- and NaOCl-based irrigation protocols. Tipping point values are indicated within each panel. MI, multiple imputation; NaOCl, sodium hypochlorite; PAI, Periapical Index.

Supplementary Table S2. Patient and tooth cluster description for within-patient clustering sensitivity analysis.

Item	Value
Number of patients (clusters)	134
Number of teeth (observations)	186
Teeth per patient — mean (SD)	1.39 (0.76)
Teeth per patient — median (range)	1 (1–4)
Distribution: 1 tooth	101 patients
Distribution: 2 teeth	18 patients
Distribution: 3 teeth	11 patients
Distribution: 4 teeth	4 patients

SD = standard deviation.

Supplementary Table S3. Sensitivity analysis accounting for within-patient clustering — Strict success.

Predictor	GLM		Robust SE		GEE Exchangeable		GEE AR1		GLMM	
	OR (95% CI)	P	OR (95% CI)	P	OR (95% CI)	P	OR (95% CI)	P	OR (95% CI)	P
Periodontal pocket >3 mm (Y vs N)	0.24 (0.10-0.54)	<0.001	0.24 (0.10-0.58)	0.002	0.27 (0.11-0.65)	0.004	0.25 (0.10-0.60)	0.002	0.20 (0.07-0.59)	0.004
Root canal closure (Y vs N)	0.26 (0.06-1.09)	0.065	0.26 (0.06-1.17)	0.079	0.28 (0.06-1.46)	0.132	0.24 (0.06-1.04)	0.056	0.21 (0.04-1.24)	0.085
Preoperative PAI (per 1-point increase)	0.76 (0.58-1.00)	0.05	0.76 (0.58-1.01)	0.055	0.73 (0.56-0.96)	0.026	0.74 (0.57-0.97)	0.030	0.72 (0.51-1.01)	0.058
ICC (random intercept) = 0.232; clusters = 134 patients (186 teeth)										

Same prespecified three-variable model as Table 4 (periodontal pocket depth >3 mm, canal obliteration, and preoperative PAI) refitted using four cluster-aware approaches. GLM = independent-observation model; Robust SE = GLM with cluster-robust sandwich standard errors clustered by patient; GEE exchangeable and GEE AR-1 = generalized estimating equation models with patient-level exchangeable or first-order autoregressive working correlation structures; GLMM = generalized linear mixed model with a patient-level random intercept. ICC was computed from the GLMM random intercept on the latent logit scale as $var(u) / [var(u) + \pi^2/3]$. ICC = 0.232 suggests moderate within-patient correlation. Cluster size distribution: 1 tooth, 101 patients; 2 teeth, 18 patients; 3 teeth, 11 patients; 4 teeth, 4 patients. OR = odds ratio; CI = confidence interval; PAI = Periapical Index. p values are two-sided.

Supplementary Table S4. Sensitivity analysis accounting for within-patient clustering — Loose success.

Predictor	GLM		Robust SE		GEE Exchangeable		GEE AR1		GLMM*	
	OR (95% CI)	P	OR (95% CI)	P	OR (95% CI)	P	OR (95% CI)	P	OR (95% CI)	P
Periodontal pocket >3 mm (Y vs N)	0.25 (0.11-0.59)	0.001	0.25 (0.10-0.65)	0.004	0.33 (0.13-0.82)	0.017	0.30 (0.12-0.71)	0.006	0.35 (0.02-6.72)	0.485
Root canal closure (Y vs N)	0.23 (0.06-0.97)	0.046	0.23 (0.05-1.12)	0.068	0.28 (0.05-1.53)	0.142	0.23 (0.05-0.96)	0.044	2.20 (0.00-1289.79)	0.808
Preoperative PAI (per 1-point increase)	0.99 (0.73-1.34)	0.958	0.99 (0.74-1.32)	0.956	0.95 (0.73-1.23)	0.678	0.96 (0.75-1.31)	0.743	0.71 (0.32-1.59)	0.404
ICC (random intercept) = 0.985; clusters = 134 patients (186 teeth)										

Same prespecified three-variable model as Table 4 (periodontal pocket depth >3 mm, canal obliteration, and preoperative PAI) refitted using four cluster-aware approaches. GLM = independent-observation model; Robust SE = GLM with cluster-robust sandwich standard errors clustered by patient; GEE exchangeable and GEE AR-1 = generalized estimating equation models with patient-level exchangeable or first-order autoregressive working correlation structures; GLMM = generalized linear mixed model with a patient-level random intercept. ICC was computed from the GLMM random intercept on the latent logit scale as var(u) / [var(u) + $\pi^2/3$]. The high ICC estimate (0.985) and very wide GLMM confidence intervals suggest model instability; therefore, GLMM estimates for Loose success should be interpreted with caution. Cluster size distribution: 1 tooth, 101 patients; 2 teeth, 18 patients; 3 teeth, 11 patients; 4 teeth, 4 patients. OR = odds ratio; CI = confidence interval; PAI = Periapical Index. p values are two-sided.

Supplementary Table S5. Patient-level baseline characteristics of the analytical cohort (n = 134 patients).

Variables	Patients (n = 134)
**Patient demographics**	
Age (years), median (range)	56 (14–89)
**Age distribution, n (%)**	
< 30 years	22 (16.4%)
30–49 years	32 (23.9%)
50–69 years	58 (43.3%)
≥ 70 years	22 (16.4%)
**Systemic medical history, n (%)**	
No systemic disease	57 (42.5%)
With ≥1 systemic disease	77 (57.5%)
Hypertension	36 (26.9%)
Diabetes mellitus	13 (9.7%)
Dyslipidemia	25 (18.7%)
Osteoporosis or osteopenia	14 (10.4%)
Cardiac or cerebrovascular disease	10 (7.5%)
Malignancy	4 (3.0%)
**Number of teeth contributed per patient, n (%)**	
1 tooth	101 (75.4%)
2 teeth	18 (13.4%)
3 teeth	11 (8.2%)
≥ 4 teeth	4 (3.0%)
Mean ± SD	1.39 ± 0.76
**Clinical characteristics, n (%)**	
Periodontal pocket depth > 3 mm	24 (17.9%)
≥ 1 tooth requiring retreatment	24 (17.9%)

Data are presented as n (%) unless otherwise specified. Age is reported at the time of treatment. Systemic disease categories are not mutually exclusive; patients with multiple conditions are counted in each applicable category. Cardiac or cerebrovascular disease includes angina pectoris, coronary artery disease, congestive heart failure, cardiac valve surgery, transient ischemic attack, and cerebral infarction. SD = standard deviation.
